# Cytostatic Effects of Polyethyleneimine Surfaces on the Mesenchymal Stromal Cell Cycle

**DOI:** 10.3390/polym14132643

**Published:** 2022-06-29

**Authors:** Anna Alba, Giusy Villaggio, Grazia Maria Lucia Messina, Massimo Caruso, Concetta Federico, Maria Teresa Cambria, Giovanni Marletta, Fulvia Sinatra

**Affiliations:** 1 Section of Biology and Genetic, Department of Biomedical and Biotechnological Sciences, University of Catania, Via S. Sofia, 65, 95123 Catania, Italy; a.alba83@gmail.com (A.A.); giusyvillaggio@gmail.com (G.V.); 2 Laboratory for Molecular Surface and Nanotechnology (LAMSUN), Department of Chemical Sciences, University of Catania and CSGI, Viale A. Doria, 6, 95125 Catania, Italy; gmarletta@unict.it; 3 Section of Biochemistry, Department of Biomedical and Biotechnological Sciences, University of Catania, Via S. Sofia, 65, 95123 Catania, Italy; massimo.caruso@unict.it (M.C.); cambrimt@unict.it (M.T.C.); 4 Section of Animal Biology, Department of Biological, Geological and Environmental Sciences, University of Catania, Via Androne, 81, 95124 Catania, Italy; concetta.federico@unict.it

**Keywords:** polymeric polyelectrolyte multilayers, bone mesenchymal stromal cells, QCM-D, cell spreading, cytostasis

## Abstract

Polyelectrolytes assembled layer-by-layer (PEMs) are commonly used as functional coatings to build-up biological interfaces, particularly suitable as compatible layers for the interaction with a biological medium, providing suitable conditions to promote or prevent cell seeding while maintaining the phenotype. The proper assessment of the biocompatibility of PEMs and the elucidation of the related mechanisms are therefore of paramount importance. In this study, we report in detail the effect of two different PEM endings, polystyrene sulfonate (PSS) and polyethylenimine (PEI), respectively, on the cell adhesion, growth, and viability of human bone mesenchymal stromal cells (MSCs). The results have shown that PSS-ended substrates appear to be the most suitable to drive the cell adhesion and phenotype maintenance of MSCs, showing good biocompatibility. On the contrary, while the cells seem to adhere more quickly and strongly on the PEI-ended surfaces, the interaction with PEI significantly affects the growth and viability, reducing the cell spreading capability, by sequestering the adhesion molecules already in the very early steps of cell–substrate contact. These results point to the promotion of a cytostatic effect of PEI, rather than the often-claimed cytotoxicity.

## 1. Introduction

Cell adhesion is a multistage process involving the attachment, spreading, and formation of stress fibers and focal adhesions at the very early steps [1], being critically dependent on surface characteristics. As is known, the bulk properties of biomaterials are very important, but their surface properties are of utmost importance, as they boost the design of functional materials and are able to drive tissue and cellular events such as protein adsorption, recolonization, adhesion, proliferation, migration, and inflammatory response [2]. Thus, controlling the surface properties of materials is a real challenge on the way to developing new generations of smart biomaterials and the related tissue engineering strategies [3].

Indeed, surface properties, such as topography, stiffness, surface free energy (SFE), roughness, and chemistry (specific chemical functionalities), have been studied in detail, showing that they all play important roles, promoting the cell response to chemical and physical cues that can be “added” or modified by surface modification [4]. One of the well-established methods to modify surface properties is to coat them by means of polyelectrolyte multilayers (PEMs), built by a layer-by-layer (LbL) technique, consisting of the alternate deposition of polycations and polyanions that self-organize on the material’s surface [5]. This methodology is simple, flexible, effective, inexpensive, and versatile and it has been applied to a large variety of biomedical devices [2,3]. Understanding the specific effect of chemically different PEMs on the initial cell attachment process, as well as analyzing adhered cells and studying the cell behavior and phenotype, may lead to a significant improvement of biomedical devices [6].

In order to better comprehend the interaction mechanisms between cells and LbL thin films, the cell accommodation on the PEM surfaces have to be studied by using a non-invasive and real-time technique with high sensitivity for the interfaces.

In this context, a technique based on the characterization of interface’s properties by using surface acoustic waves as sampling tools, i.e., quartz crystal microbalance with dissipation monitoring (QCM-D), can be employed. This technique, indeed, has been widely used to study the interaction process of “soft” matter thin layers with solid surfaces, including the formation of interfaces with ultrathin polymer films, biomolecules, nanoparticles, vesicles and, in general, biological systems [7,8,9]. Moreover, the technique has been successfully extended to the study of cell–substrate interaction events, owing to the specific sensitivity of the technique to the viscoelastic changes occurring in the wall region of the cells interacting with the sensor surface [10,11,12].

The aim of the present work was to study the early steps of cell–surface interaction using two different PEM coatings, to understand the effect of surface physicochemical properties on cell adhesion, morphology, and proliferation. In particular, we studied the early events of the interaction between mesenchymal stem cells isolated from human bone marrow (MSCs) and PEMs of polystyrene sulfonate (PSS) (bearing negatively charged groups) and polyethyleneimine (PEI) (positively charged polymer), both assembled with the LbL deposition technique. It is worth mentioning that while PSS is known to have a good biocompatibility with the biological medium, the literature has reported that polyethyleneimine (PEI), on the other hand, exhibits some form of “toxicity” towards various cell lines [13].

In this context, the mechanical response of the cells to the two PEMs was studied by using the QCM-D technique, while the differential cell response was investigated by means of thorough biological assays, consisting of cell viability tests, morphological changes due to the rearrangement of the actinic and tubulin cytoskeleton, and tests evaluating the formation of focal adhesion complexes. Finally, mitochondrial activity was also examined through the use of a fluorescent probe, sensitive to the variation of the mitochondrial membrane potential.

Our results showed a remarkable difference between the two PEMs on the cells in cytoskeleton reorganization and related different effects on cell adhesion, growth, and metabolism.

More specifically, while on the PSS-ended surface the cells retain the MSC phenotype and show good compatibility, on the PEI-ended surface the cells adhere faster and strongly, blocking the cell growth and spreading, owing to the random and peripheral formation of many focal adhesion sites in connection with the sequestering of the adhesion molecules in the initial cell–substrate contact area. Overall, these results point to a cytostatic effect of PEI-ended coating, rather than the often-claimed cytotoxicity effect, actually mostly reported for PEI in solution, instead of compact anchored layers.

## 2. Materials and Methods

### 2.1. Polyelectrolyte Multilayers

Polyelectrolyte solutions were prepared as follows: PEI (MW 750,000) and PSS (MW 70,000) were purchased from Sigma, Milan, Italy and solubilized in a 0.15 M NaCl solution at a concentration of 1 mg/mL. Before PEM deposition, the surfaces were irradiated with UV-O_3_ for 30 min at atmospheric pressure in a Jelight Instruments apparatus (Jelight Company Inc., Irvine, CA, USA) (λ_ex_ of 185 and 254 nm) to remove any carbon moieties, washed extensively with ultrapure water, and dried with blown nitrogen. Then, the deposition of the PSS (−) and PEI (+) polymeric layers on the wells and on the slides to be used for our study using the LbL technique was carried out. Simple immersion in the PEI or PSS solution for 15 min was enough to achieve a complete layer of each polyelectrolyte: PEMs were built-up on the surfaces by alternating PEI and PSS layer deposition to obtain a PEI-PSS film with an anionic surface, and a PEI-PSS-PEI film with a cationic surface. The obtained multilayers were completely dried and incubated for 2 h with antibiotic–antimycotics andm after two washings with PBS, experiments were started by adding the cell suspension to each sample. Culture plates or glass slides without preliminary treatment with PEMs were used as control samples. For clarity, the cell-facing outer layer is used throughout the text as a shorthand for multilayers, with PEI-ended indicating the PEI/PSS/PEI multilayer and PSS-ended indicating the PEI/PSS multilayer.

### 2.2. Cell Culture

Mesenchymal stromal cells were obtained from human bone marrow aspirates from healthy donors after they had given their informed consent. hBMSCs were seeded in 75 cm^3^ T flasks and expanded in minimum essential alpha medium (αMEM) supplemented with L-glutamine, nucleosides and Earle’s salts, antibiotic–antimycotic (10,000 U/mL penicillin, 10,000 μg/mL streptomycin, 25 μg/mL Fungizon), 10% fetal bovine serum (FBS), and 100 μm of ascorbic acid (Sigma-Aldrich, St. Louis, MO, USA). The culture flasks were maintained in a 37 °C incubator, with a humidified atmosphere of 5% CO_2_. Medium was changed after 3–4 days to remove non-adherent cells and replaced with fresh medium. When the cell monolayer was approximately 80% confluent, the cells were detached by trypsin/EDTA (0.05%/0.2% *w*/*v*) and seeded in T25 flasks with complete αMEM, and 10% FBS. Flow cytometric analysis revealed positivity for CD105, CD90W, and CD73 and not for CD34, CD14, and Gly A, [14]. All products were purchased from GIBCO (Life Technologies, Carlsbad, CA, USA).

### 2.3. Scanning Electron Microscopy

MSCs were cultured on PSS-ended or PEI-ended round glass coverslips. At the prefixed experimental time (24 h), cells were fixed in 2% glutaraldehyde in 0.1 M Na cacodylate buffer (EMS), pH 7.4, and then post fixed in 1% osmium tetroxide (EMS) in the same buffer. The samples were dehydrated in graded ethanol followed by critical point drying (Emscope CPD750), mounted on stubs, and sputter coated with gold (Sputter Coater, Polar SC7640, Quorum Technologies, East Sussex, UK). Cell morphologies were observed with a field emission scanning electron microscope (FESEM Hitachi S4000, Fukuoka, Japan).

### 2.4. Cytoskeleton and Adhesion Molecules

*Direct immunofluorescence—*The actin organization of MSCs (1 × 10^4^ cells) growing for 3, 6, and 24 h on round glass coverslips coated with the PSS-ended or PEI-ended multilayer was analyzed by direct immunofluorescence using fluorescein-labelled phalloidin (Sigma Aldrich, Italy). The samples were fixed with 3% paraformaldehyde/PBS containing 2% saccharose, permeabilized with 0.5% Triton X-100/PBS for 5 min, and incubated with FITC-phalloidin (20 µL/mL PBS) for 30 min at 37 °C.

*Indirect immunofluorescence—*IF was used to reveal tubulin, integrin α1, integrin α5, FAK-pY397, and paxillin pY31. The cells were initially treated as set out above, but after permeabilization they were incubated in blocking buffer (BSA 5% in PBS) for 30 min and with the primary antibody (1 h). Samples were then washed in PBS and incubated with red or green fluorescent secondary antibody goat anti-rabbit IgG Alexa Fluor 594/488 (2.5 µg/mL) (Immunological Science, Rome, Italy) for 1 h at room temperature. After two washes with PBS, the samples were allowed to dry and were mounted with Fluoro Gel with DAPI (EMS). Fluorescence was observed with an Olympus BX50 fluorescence microscope equipped with a DC500 camera (Leica, Wetzlar, Germany). pFAK and p-paxillin were observed using a laser scanning confocal microscope (Zeiss LSM 700, Oberkochen, Germany). Green and blue signals were detected with laser light at 488 nm and 405 nm, respectively. All acquisitions with the laser scanning confocal microscope were performed using ZEN-2010 software.

*α5 Integrin and microfilament double labeling*—To better highlight the relationship between microfilaments and integrins, double labeling was carried out with primary antibodies for the cytoplasmic subunits of the two integrins and FITC-phalloidin. Microfilaments were stained after incubation with Alexa Fluor 594 (Thermo Fisher, Waltham, MA, USA).

The following antibodies were used after dilution in BSA 1% in PBS: mouse monoclonal anti α-tubulin (1:2000 dilution) (clone B 5-1-2; Sigma-Aldrich, Italy); integrin α5 subunit (cytoplasmatic) (1:1000 dilution); integrin α1 subunit (cytoplasmatic) (1:500 dilution) (Immunological Science, Rome, Italy); phospho-paxillin (Tyr31) antibody (44-720G) (1:200 dilution); and phospho-FAK (Tyr397) recombinant rabbit monoclonal antibody (clone 31H5L17) (1:200 dilution) (Invitrogen, Waltham, MA, USA).

### 2.5. Quartz Crystal Microbalance with Dissipation Monitoring (QCM-D)

Measurements of adsorption kinetics were performed by using a quartz crystal microbalance with dissipation monitoring (QCM-D) instrument (Q-Sense AB, Gothenburg, Sweden) with AT-cut gold crystal sensors. The simultaneous measurements of frequency, f, and energy dissipation, D, were performed in the fundamental resonance frequency (*n* = 1, i.e., *f* = 5 MHz) and the overtones (*n* = 3, 5, 7, and 9 corresponding to *f* = 15, 25, 35, and 45 MHz, respectively). The resolution in *F* and *D* was ± 0.1 Hz and 1 × 10^−7^, respectively. Each QCM-D experiment started with the sensor running on PBS (outgassed with 30 min sonication), then the addition of cells and, after 180 min, the exchange of the solution being measured with PBS to check both the desorption and stability of the adsorbed layer. All the experiments were performed in water at 37 °C and the flow rate was 150 μL min^−1^. Three replicas for each experiment were performed for data reproducibility.

### 2.6. Cell Viability and Cell Cycle Assessment

MSCs were seeded (1 × 10^4^ cells/well) on a 24-well plate previously coated with PEMs, and incubated for 3 h, 24 h, 48 h, 72 h, and 120 h at 37 °C. Mitochondrial activity was measured by the MTT assay, incubating the cells with 0.05 mg/mL/well 3-(4,5-dimethylthiazol-2-yl) 2,5-diphenyltetrazolium bromide salt (MTT; Sigma Aldrich, Italy) in serum free medium. After 2.30 h of incubation at 37 °C, formazan salts, produced by succinate dehydrogenase activity in live cells, were solubilized with 0.1 M isopropanol//HCl and quantified spectrophotometrically by a Cary 50 (Varian, Palo Alto, CA, USA). The values obtained are expressed as corrected optical density (Δ OD: λ570−λ650). All samples and experiments were carried out in triplicate. Evaluation of the cell cycle was performed using the Muse^®^ Cell Cycle Kit (Luminex Corporation, Austin, TX, USA) by the Guava^®^ Muse^®^ Cell Analyzer following the manufacturer’s instructions. Briefly, cells were trypsinized, washed, and resuspended in fixing solution with 70% ethanol and then stained with cell cycle reagent containing propidium iodide (PI). On average, 5000 events were acquired per sample. Data were generated by the Muse^®^ Cell Cycle software module.

### 2.7. ATP Evaluation and Mitochondrial Transmembrane Potential (ΔΨ)

Cellular ATP content was measured using a firefly luciferase-based ATP assay kit (ATPlite 1 step Luminescence, Perkin Elmer, Waltham, MA, USA) according to the manufacturer’s instructions. Samples were lysed and quantified after 3 h, 6 h, and 24 h of culture. The light emission was directly proportional to the concentration of ATP and is expressed in counts per second (CPS). The mitochondrial transmembrane potential was determined by fluorescence microscopy after incubation with JC-1 (5,5′, 6,6′-tetrachloro-1,1′, 3,3′-tetraethylbenzimidazolylcarbocyanine iodide; Molecular Probes, Eugene, OR, USA). MSCs were seeded on glass slides and PEI-ended slides for 3, 24, and 48 h, incubated with 0.1 μM JC-1 fluorescence probe for 30 min, and washed three times with PBS. Mitochondrial membrane potential was observed with an Olympus BX50 fluorescence microscope (Olympus Italia, Segrate, Italy). The green JC-1 signals appeared at 485/535 nm, and the red signals appeared at 590/610 nm. Some experiments were performed to test the effect of exogenous ATP on cell viability, actin cytoskeleton, and ATP content of MSCs on PEI-ended surfaces. The culture medium was supplemented with 20 µM, 10 µM, and 5 µM of ATP (Sigma Aldrich, Italy) and, after intervals of 3 and 24 h of incubation, samples were prepared for MTT assay, ATP content, and FITC-phalloidin assay. After 72 h of incubation, the medium was replaced with fresh α MEM containing the different concentrations of ATP and incubated for another 3 h. All experiments were carried out in triplicate.

### 2.8. Statistical Analysis

Statistical analysis data are expressed as mean ± standard deviation (S.D.) of three independent experiments (i.e., biological and technical triplicates). We evaluated the statistical significance of these data by applying the one-way ANOVA test, as described in the figure legends.

## 3. Results and Discussion

### 3.1. Morphological Analysis

**Figure 1:**
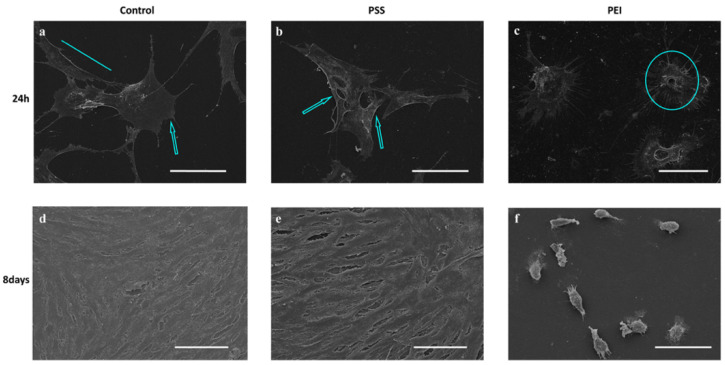
Scanning electron micrographs (SEM) of MSCs grown for 24 h and 8 days on glass (**a**,**d**), PSS-ended (**b**,**e**), and PEI-ended (**c**,**f**) slides. Scale bar: control and PSS, 80 µm; PEI, 40 µm. Cyan line, arrows, and circle show cell morphology on three different substrates.

The initial MSC attachment and spreading were visualized using scanning electron microscopy. The morphology of cells after 24 h of adhesion on PEMs was different with respect to the control as shown in Figure 1. Indeed, the cells adhering to the PSS-ended surface had a regular and elongated morphology (Figure 1b), similar to the control (Figure 1a), with long pseudopodia suggesting that they had begun to spread, as shown by the cyan arrows and line, while MSCs on the PEI-ended substrates (Figure 1c) maintained a more rounded morphology with a flat cytoplasmic edge from which thin filaments branched off towards the substrate, as shown by the cyan circle.

After 8 days, cells adhering to the PSS-ended films formed a confluent monolayer (Figure 1e), while on the PEI-ended substrates, they did not form a monolayer, retaining their initial rounded shape and showing an evident irregular cellular morphology (Figure 1f). Overall, the data suggest a remarkable dependence of cell response on the chemical nature of the two surfaces.

### 3.2. Cytoskeleton and Adhesion Molecules

#### 3.2.1. Microfilaments and Microtubules

**Figure 2:**
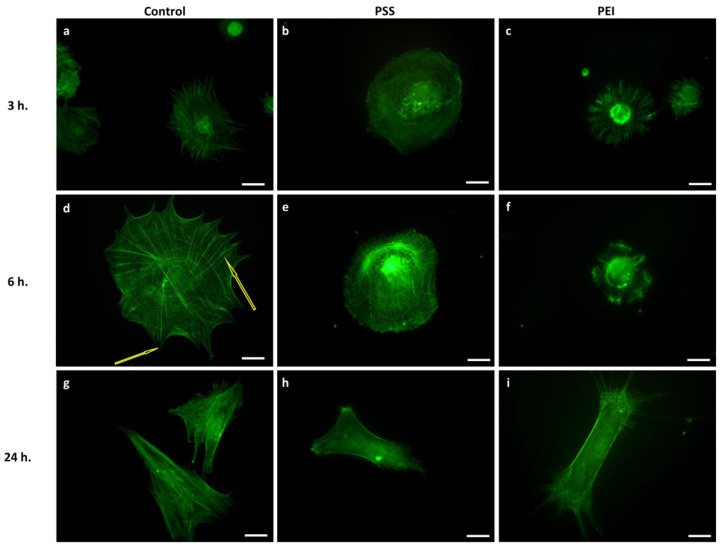
Representative images of the cytoskeleton structure of MSCs after 3 h, 6 h, and 24 h of culture time on glass slides (**a**,**d**,**g**), PSS-ended slides (**b**,**e**,**h**), and PEI-ended (**c**,**f**,**i**) slides. Green represents a FITC-phalloidin stain of the actin cytoskeleton. Scale bar: 5 µm. Yellow arrows show stress fibers and filopodia formation.

We analyzed changes in the cell shape and cytoskeletal organization of microfilaments and microtubules during the early events of cell adhesion (3 h, 6 h, 24 h) on polyelectrolyte multilayer films compared with MSCs seeded on control surfaces. Direct immunofluorescence results showed that a noticeable change in actin distribution, cell morphology, and size occur during the adhesion and spreading processes of MSCs (Figure 2). Again, on the PSS-ended slides, cells showed an actin distribution and organization in microfilaments very similar to the control, even if the adhesion and spreading processes seemed to proceed more slowly.

After 3, 6, and 24 h, they assumed an elongated morphology with regular and numerous stress fiber formation (Figure 2b,e,h). On PEI-ended slides, the MSCs, after 3 and 6 h (Figure 2c,f), instead showed a rounded morphology with a distribution of fluorescent protrusions that were extended radially in the periphery. After 24 h some cells spread on the substrate and displayed long protrusions and numerous filopodia, without stress fibers, but with an evident actin cortex (Figure 2i). These results revealed that PEI had a strong, hindering effect on the correct formation of stress fibers, expected, in turn, to severely affect the cell cytoskeletal functions.

**Figure 3:**
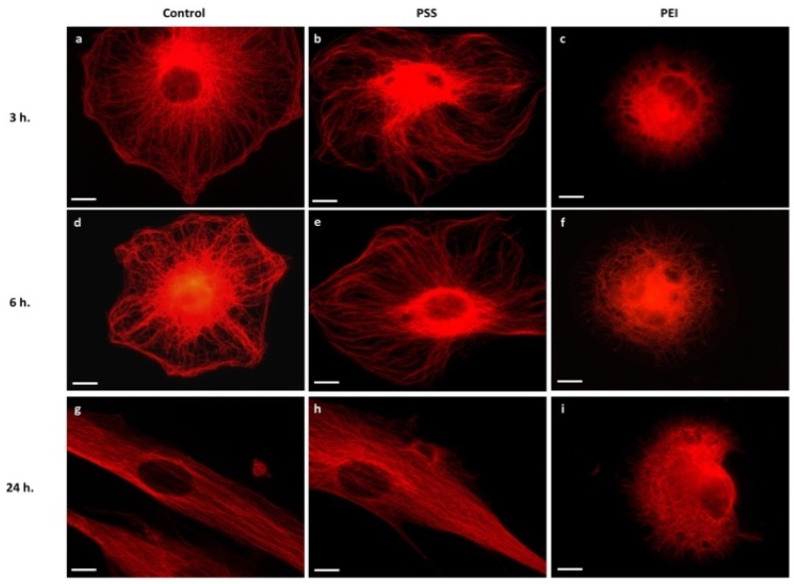
Representative images of cytoskeleton structure of MSCs after 3 h, 6 h, and 24 h of culture time on glass slides (**a**,**d**,**g**), PSS-ended slides (**b**,**e**,**h**), and PEI-ended (**c**,**f**,**i**) slides. Red represents anti α-tubulin and Alexa Fluor 594 stain of microtubules. Scale bar: 2 µm.

As far as motor-driven and polymerization-dependent forces can also be mediated by other cytoskeletal systems, such as microtubules and their associated protein, and cytoskeletal proteins, in view of their importance for intracellular transport, mitosis, and migration [15,16,17,18], we have also investigated the variation of microtubule organization and distribution during the early stages of cell adhesion on PEMs (Figure 3). Again, on PSS-ended substrates the MSCs exhibited a thin and well dispersed microtubular network, with a higher fluorescence intensity around the nucleus (Figure 3e), and at 24 h (Figure 3h) they showed an elongated shape of healthy cells. At variance with this, a significant alteration in the microtubule arrangement in the cells on PEI-ended substrate was observed, the microtubules being concentrated and compact, with a felt-like structure, in the perinuclear area and in the cytoplasm, aggregating into a thicker microtubular network (Figure 3c,f,i).

As a consequence of this failure to organize the actin cytoskeleton, the morphology of the cells on PEI-ended films did not change, remaining rounded and compact.

These results confirm the remarkable effect of the chemical nature of the substrates, for these two PEMs films, on the behavior of the cytoskeletal elements and in particular on their correct assembly. The hindered formation of the appropriate cytoskeletal structures will be responsible for the compromised correct cell morphology and the related cellular functions [19]. It should be stressed that the reported data provide a first explanation of the cell morphologies revealed by means of the SEM analysis above (Figure 1).

#### 3.2.2. Integrins

**Figure 4:**
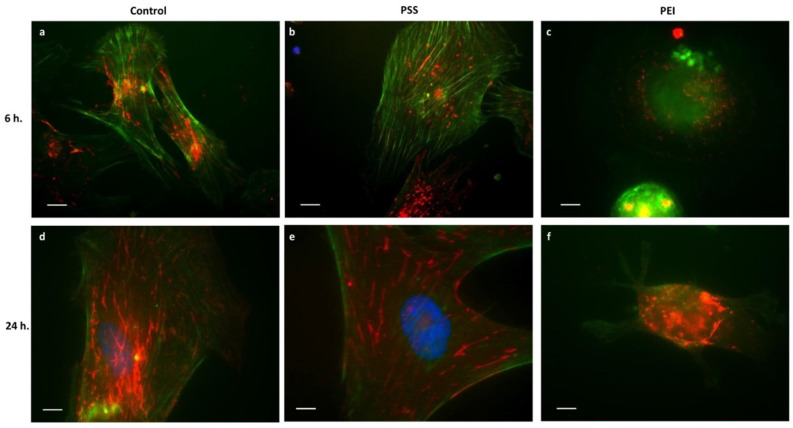
Distribution of Integrin α5ß1 (red fluorescence) and actin (green fluorescence) in MSCs after 6 h and 24 h of culture time on glass slides (**a**,**d**), PSS-ended slides (**b**,**e**), and PEI-ended (**c**,**f**) slides Blue represents DAPI stain of nuclei. Scale bar: a and b = 5 µm.

Adhesion molecules, such as integrins, act primarily to provide localized signals rather than to support physical attachment, and they are directly connected to the actin cytoskeleton with the activation of a signaling cascade that regulates many cellular functions [20,21]. In view of this, it is important to investigate their localization and distribution during the early steps of cell–PEM interactions. Cell–material interactions are mediated by a layer of proteins that in our in vitro experiments are present in the FBS 10% culture medium [22] and absorbed at the cell–substrate interface. As fibronectin and collagen are the two proteins most expressed by MSCs [23], we analyzed the α5ß1 (fibronectin receptor) and α1ß1 (collagen receptor) integrins distribution, and the results are shown in Figure 4. A normal expression of integrins in all samples, but no translocation in the PEI-ended sample, was found at 24 h (Figure 4). The cell–surface contact in reference samples induced integrin clustering (dot-like fluorescence) that shifted towards the cell periphery with a rod-like organization, finally yielding newly assembled actin filaments (Figure 4a,d). As for the PSS-ended surface (Figure 4b), at 6 h the α5ß1 integrin showed clustering and translocation towards the cell periphery, running parallel to the actin cytoskeleton, indicating a good adhesion and an optimal spreading performance at 24 h (Figure 4e). At variance with this, on the PEI-ended films, the integrins remained punctiform, with a spot-like fluorescence localized and dispersed in the central area and in the periphery of the cell (Figure 4c), which, after 24 h of adhesion, produced a disordered clustering, but without peripheral translocation, pointing to a strong cell adhesion but a very limited spreading (Figure 4f), again matching closely with the morphology findings (see Figure 2).

It should be stressed that the strong adhesion of the cells to the PEI-ended surfaces, inhibiting the spreading, could have “physically” prevented the translocation of the integrins through a mechanism involving the lack of actin polymerization, caused by ATP deficiency (see below), which is, thus, unable to generate the traction force needed for the displacement (i.e., translocation) of the integrin/microfilament complexes.

It is known, in fact, that a correctly polymerized actin cytoskeleton is a necessary requirement for the assembly of integrin-dependent focal processes [24] giving rise to mechano-transduction forces involved in various physiological and pathological processes [25].

Finally, as for the α1ß1 integrin (Figure S1), this was found to be more uniformly diffused in the central area of the cell body after 6 h of adhering to all the surfaces; no significant differences were found among the analyzed samples.

Overall, the reported results show that, on the one hand, the numerous membrane specializations (filopodia, lamellipodia, and blebs) induced by PEI-ended surfaces in mesenchymal cells could indicate a good degree of cell adhesion, while on the other hand, the absence of microfilaments and the lack of translocation of integrins severely limit the correct spreading process, producing the characteristic rounded cell morphology.

#### 3.2.3. Focal Adhesion Kinase and Paxillin

Focal complexes (FCs), with a characteristic dot-like shape, evolve in focal adhesions (FAs), with a rod-like morphology, being associated with long actin and myosin fibers located peripherally and near the perinuclear region of the cell [26].

The formation of FAs and stress fibers is accompanied by the phosphorylation of many specific proteins, including FAK, paxillin [27], and the FAK-SRC complex that promotes the cell cycle progression in normal and cancer cells [28,29,30,31,32].

**Figure 5:**
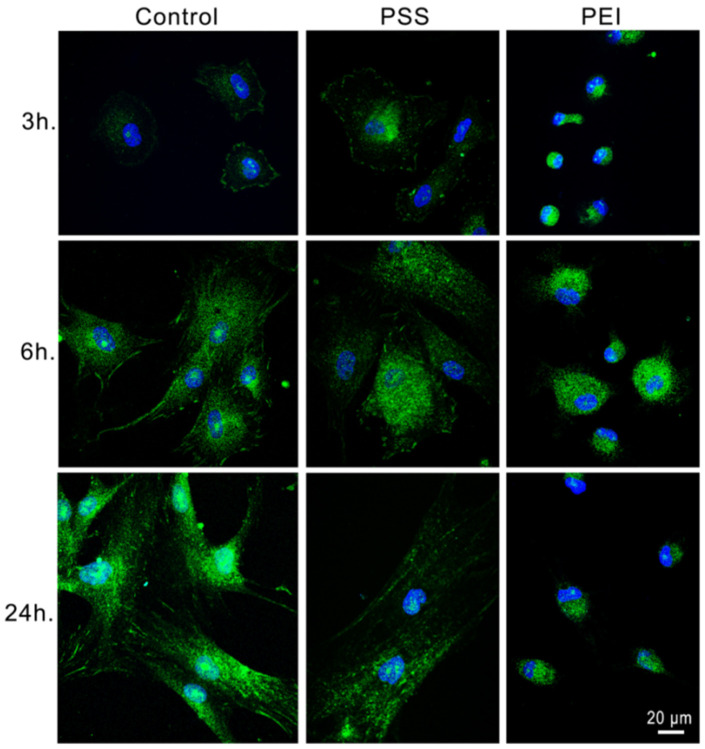
Representative images of MSCs after 3 h, 6 h, and 24 h of culture time on glass slides, PSS-ended slides, and PEI-ended slides. Green represents FITC-conjugated antibody stain of FAK, blue represents DAPI stain of nuclei. Scale bar: 20 µm (for all the images shown).

Accordingly, the analysis of the localization of phosphorylated FAK and paxillin and their punctiform or rod-like organization in adhesion cells provided the degree of maturation of focal adhesions for the various substrates here investigated. Indeed, in the control and in the PSS-ended substrates, during the first few hours of MSC adhesion, a well-defined maturation of the FCs in FAs was observed, with the characteristic rod-like organization and peripheral localization of pFAK (Figure 5), both indicating that a correct assembly of the adhesion machinery occurred.

On the other hand, for MSCs on PEI-ended surfaces, the phosphorylated FAK appears punctiform and uniformly distributed in the central area of the cell body, but not in the periphery, where only a lower amount of it seems to have translocated.

Also, more pFAK was observed in cells attached to glass or PSS-ended surfaces with respect to those seeded on a PEI-ended multilayer.

As is known, the biological function of phosphorylated PXN (pPXN) is to regulate cell spreading and motility through the organization of the actin cytoskeleton, by generating binding sites for other proteins, promoting the mechanical binding to the actin cytoskeleton, and, finally, producing the event cascade to regulate proteins involved in the management of numerous cellular functions [33]. Therefore, the previous observations on the formation of the actin cytoskeleton have to be confirmed by the careful analysis of the fate of pPXN.

In particular, we found that the cells on the control substrates at 3 h showed a dot-like paxillin structure, also evident near the nucleus, finally evolved at 24 h to a dominating rod-like structure as a result of the cell spreading.

**Figure 6:**
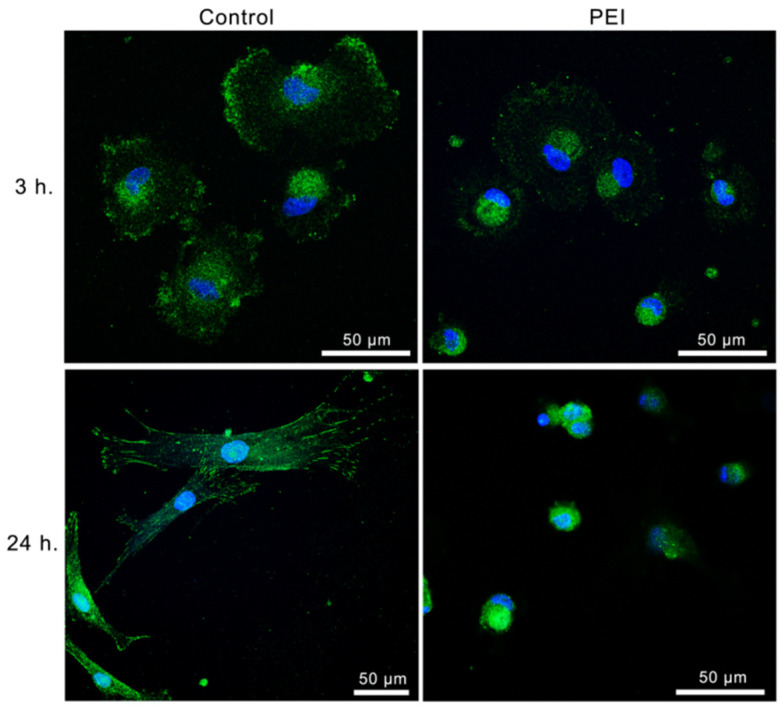
Representative images of MSCs after 3 h and 24 h of culture time on glass slides and PEI-ended slides. Green represents FITC-conjugated antibody stain of FAK, blue represents DAPI stain of nuclei. Scale bars: 50 µm.

As for MSCs on PEI-ended surfaces, it was found that from 3 to 24 h of cell incubation (Figure 6) the pPXN gradually extended towards the cell periphery following the final localization of the FAK.

More specifically, after 3 h pPXN was predominantly localized near the nucleus and in the peripheral adhesion zone of the mesenchymal cells, as in the control (although with less evident fluorescence). However, at 24 h pPXN was found to concentrate around the nucleus, while the previous adhesion zones were no longer appearing and the cells did not spread.

These alterations in the maturation of the adhesion plaques on PEI-ended substrates are perfectly congruent with the failure in the peripheral translocation of pFAK and pPXN, which indeed stops at the dot-like stage, and reflect the disarrangement of actin and microtubules disorder, corresponding to the patterns above reported for integrins on the same substrates.

Finally, in view of the direct connection between the adhesion molecules and the regulation of cellular functions, highlighted by the ability of FAK to translocate to the cell nucleus, further regulating cell proliferation, we have characterized the distribution of pFAK in the MSCs on the three substrates (control, PSS-ended, and PEI-ended).

We have observed that, for control and PSS-ended films, pFAK is mainly localized in FAs and the cell cytoplasm, only in small part being able to translocate to the nucleus. On the contrary, on PEI-ended surfaces there is also a translocation of FAK into the nucleus (Figure 5), but without any progression in the cycle (block in phase G2/M, see Table 1) and hindering the further proliferation, as far as the action carried out by FAK at the level of the nucleus consists of activating the mitogen-type signals that prompt the cell proliferation, following well-studied pathways [34,35].

### 3.3. Monitoring Cell Behavior with QCD-D Experiments

The quartz crystal microbalance with dissipation monitoring (QCM-D), which is commonly used to detect in situ and real time biomolecular interactions with surfaces, was used here to study the interaction between cells and interface to understand the underlying mechanisms at an early stage of the cell–surface interaction.

However, it is necessary to make some theoretical considerations prompting the further interpretation of the results. Generally, for the adsorption of a rigid film, the Sauerbrey equation can be applied, since it assumes the proportionality between the frequency shift and the deposited mass. This assumption, however, is not valid for the adsorption of a sub-monolayer of cells. The analysis of the response of a QCM-D sensor, when interacting with a cell, is complex, owing to the fact that the decay length for a shear wave in liquid or in dense fluid phases is considerably shorter than cell thickness (i.e., about 250 nm in water with a density of ~1 g/mL) and exponentially less for fluid phases of greater density, as a cell may be considered. This means that the quartz sensor may sample only a fraction of the whole cell’s mass, yielding a correspondingly low frequency shift. Therefore, each adhered cell can be considered as an added effective mass, much lower than the real cell mass uptake, thus preventing the straight use of the frequency shift. However, the energy dissipation (recorded as D shift) can be related to the mechanical properties of the cell membrane, in contact with the quartz sensor, and the more or less thick fluid portion in the cytoplasm overlaying the membrane. Generally, it is possible to describe the cell–substrate interaction as follows: the initial cell–substrate physical contact leads to the first QCM-D response, i.e., a decrease of the frequency shift, cell spreading, and modification of the adhesion properties affects the signal and, finally, changes in the cytoskeleton of the cells, which influence their rigidity, involve the dissipation factor. In particular, changes in dissipation give a qualitative measure of how rigidly the cell adheres to the substrate, i.e., an increase in dissipation suggests a relatively softer cell layer [10]. The relevant information for this complex system is provided by the ΔD/ΔF ratio (D–F plot), which, in fact, does not depend on the apparent cell mass, as it represents the energy loss per unit of attached mass. In particular, the D–F plots reflect the viscoelastic properties of the “normalized” mass attached to the sensor, involving, in turn, the effect of the shape and internal structure of the cells (i.e., the spreading factor). Accordingly, QCM-D measurements provide a qualitative diagnostic of the internal structure and spread of the cells, i.e., of the rigidity of the cell region in contact with the quartz sensor as a function of the mechano-transduction forces relative to the organization and distribution of actin and their change during early adhesion steps.

**Figure 7:**
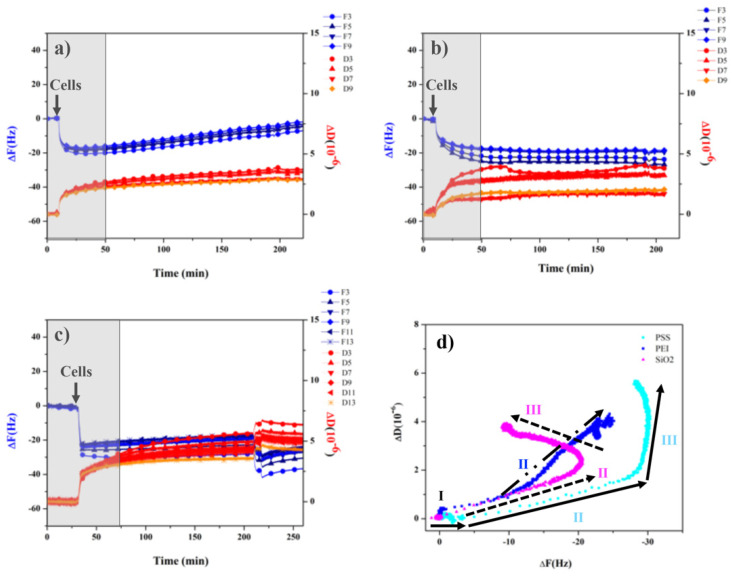
QCM-D frequency and dissipation versus time curves for cells added to (**a**) SiO_2_, (**b**) PEI, and (**c**) PSS. (**d**) Plot of ΔD as a function of ΔF of cells on SiO_2_ (magenta), PEI (blue), and PSS (cyan).

In the present work, we simultaneously measured the frequency and dissipation shifts occurring during the very early stage (3 h) of cell adhesion on PEMs and reference SiO_2_ surfaces (Figure 7). Following the cell suspension injection, all substrates showed an immediate decrease of the resonance frequency (ΔF), indicating an apparent cell mass adhesion to the surface of the sensors, while the dissipation (ΔD) increased for all samples, in agreement with the sampling of more or less viscoelastic layers in contact with the surfaces.

The SiO_2_ surfaces (Figure 7a) showed a particular trend, consisting of the complete reversal of the initial apparent mass uptake during the very early incubation time (180 min), qualitatively suggesting that an attachment–detachment process was occurring, from the initial cell adhesion event (in the first 50 min) read as a loss of frequency, followed by the re-increase in frequency, which suggests cell detachment. It must be stressed that the frequency data are in no way quantitatively meaningful, but are merely indicative of the qualitative trend of the cell–sensor interaction.

For PEI-ended surfaces (Figure 7b), the apparent cell mass adhesion (associated with a stable frequency loss) still occurs gradually; however, it remains stable over time also after a washing step with PBS. Moreover, dissipation increases in a stable way, yielding slightly spread overtones, diagnostic of a more viscous response of the cell adhered regions with respect to the SiO_2_ case. The ΔD data indicate that the cells are attached on PEI-ended substrates, hindering their spreading on PEI substrates, in agreement with the observed rounded morphology and the formation of an evident rigid actin cortex (see Section 3.1 and Section 3.2 above).

At variance with this, for cells on PSS-ended surfaces (Figure 7c), an abrupt frequency decrease was observed, qualitatively diagnostic of a diffuse cell adhesion to the substrate, followed by small frequency changes. The related dissipation was found to be significantly higher than the one for SiO_2_ and PEI, suggesting that in this case, the contact sites were more viscous than in those cases and that the cells are substantially more mobile on the surface, prompting the efficient cell spreading process responsible for the best cell seeding on PSS-ended substrates [36]. Finally, the ΔD/ΔF plots for the cell adhesion to the three surfaces are reported in Figure 7d. In particular, for each substrate, different regions can be seen: region I, common to all substrates, is characterized by a slightly increasing ΔF, while ΔD does not change, suggesting that the cells are merely adhered to the surface by gravity. Furthermore, in region II, for the SiO_2_ substrate (magenta triangles in the figure) ΔF decreases and ΔD increases steeply, suggesting the occurrence of firm cell attachment, while, in region III, a greater mass removal, with only a smaller increase in dissipation compared to region II, is observed, indicating the detachment of loosely attached cells.

For PSS-ended (negatively charged surface, cyan circles), a very similar behavior for region II is observed, whereas in region III, ΔF continues to gradually decrease and ΔD continues to increase. This overall behavior has been related to ECM remodeling and the related cytoskeletal changes, leading to an increase in the rigidity of the cellular membrane, associated with the establishment of strong cell focal adhesion points [1]. Thus, the overall ΔD/ΔF plot indicates that for the PSS-ended surface there is an effective and positive cell adhesion and spreading. Finally, on the PEI surface (positively charged surfaces, blue squares), the slope of region II becomes steeper because cells adhere strongly to the surface, while region III is missing, indicating that ECM remodeling does not occur in these early cell adhesion stages, that is, within 3 h of incubation.

### 3.4. Cell Viability and Cell Cycle

**Figure 8:**
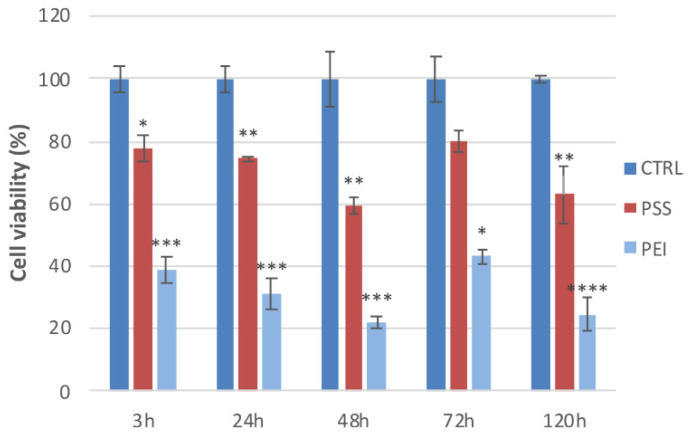
Cell viability: MSCs show different viability towards PSS and PEI after 3, 24, 48, 72, and 120 h. Results are presented as mean ± standard deviation (S.D.) of three independent experiments and normalized with respect to the control untreated cells (* *p* < 0.05, ** *p* < 0.01, *** *p* < 0.001, and **** *p* < 0.0001 versus CTRL, one-way ANOVA).

In order to determine whether PEMs affect cell viability, we analyzed their effects on MSCs for 120 h by MTT assay (graphic in Figure 8). Indeed, cell viability is reduced for both polyelectrolytes, with respect to the control, after 3 h to 78% and 39%, for PSS-ended and PEI-ended, respectively, and to 59% and 22% (again, for PSS and PEI) after 48 h.

The cell cycle, analyzed for 48 h and 6 days by flow cytometry, is shown in the Table 1.

After 48 h of treatment with PSS-ended, we did not observe significant changes in the cell cycle with respect to the control, but a reduction of the G2/M phase was observed with PEI-ended (5.1% decrease). After 6 days of treatment, we observed a reduction of the G0/G1 phase and a slight increase in phases S and G2/M in cells cultured on the PSS-ended slides. In the same conditions, we observed on PEI-ended slides a conspicuous reduction of the G0/G1 phase (16.4% decrease) and an increase of the G2/M phase (31.9% increase). It is possible that there is a stimulating effect of PEI-ended on the progression of the cell cycle from the G0/G1 phase towards the G2 phase and mitosis.

These results confirm that PEI worsens cell viability and the cell cycle, in agreement with the abnormal cell morphology that cells show on PEI-ended samples.

The strong reduction of the G0/G1 phase (Table 1) could highlight a blocking at the mitotic or pre/mitosis (G2) phase of the cell cycle, with the consequent decrease of cell viability observed in cells cultured on PEI slides as reported in the MTT results, that could be assigned to the strong adhesion of cells on the surface.

Cell cycle progression is regulated by a series of signals from the extracellular matrix. The cytoskeletal organization of a cell can have a direct influence on the progression of the cell cycle, as demonstrated in fibroblasts with a not well-structured cytoskeleton [37]. The early variations in the phosphorylation of FAK and paxillin and their correct assembly are a necessary condition to stimulate the transition from the G1 phase to the S phase of the cell cycle following adhesion, through the activation of the RAS-MAP kinase cascade (proteins activated by mitogens) and consequent activation of the retinoblastoma (RB) protein and induction of cyclin D [38]. Strong integrin-mediated attachment to a substrate serves as a checkpoint for cell cycle progression, and there is evidence that signals arising from focal adhesion (FA) directly communicate with the pathway that regulates cell proliferation. The FAK, which is associated with focal adhesion proteins, interacts directly with the GRB2 adapter protein. Since GRB2 is linked to Ras, this explains the anchorage dependence of the cellular response to growth factor-initiated mitogenesis [39].

### 3.5. ATP Evaluation and Mitochondrial Transmembrane Potential (ΔΨ)

**Figure 9:**
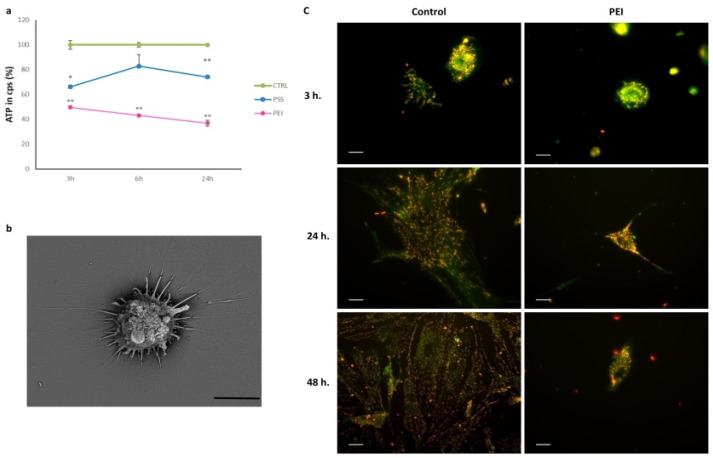
Effect of the PEI layer on (**a**) ATP content and (**b**) ultrastructure and mitochondrial transmembrane potential (ΔΨ) on MSCs. (**a**) Values (cps) are derived from the average of three experiments and expressed as percentages referring to the control; * *p* < 0.05 and ** *p* < 0.01 versus CTRL (one-way ANOVA). (**b**) Scanning electron micrography; scale bar: 10 µm. (**c**) ΔΨ analyzed with JC1 red represents J-aggregates, i.e., active mitochondria; scale bar: 10 µm.

In light of the results obtained from cells on PEI-ended slides and to better understand their behavior, we evaluated the content of ATP and the mitochondrial transmembrane potential (Figure 9). Membrane permeability plays a key role in regulating the traffic across the membrane; its alteration generates a deregulation, leading to the hydrolysis of molecules essential for cell survival and for numerous biological functions, such as ATP.

The chemiluminescence ATP quantification assay showed a clear decrease in intracellular ATP in the PEI-ended samples compared to the PSS-ended ones (Figure 9a). After 3 h of adhesion on the PEI-ended slide, MSCs showed a decrease of intracellular ATP by about 50%, which remained constant at subsequent time points, unlike for PSS, in which a resumption of cellular metabolic activity was evident.

**Figure 10:**
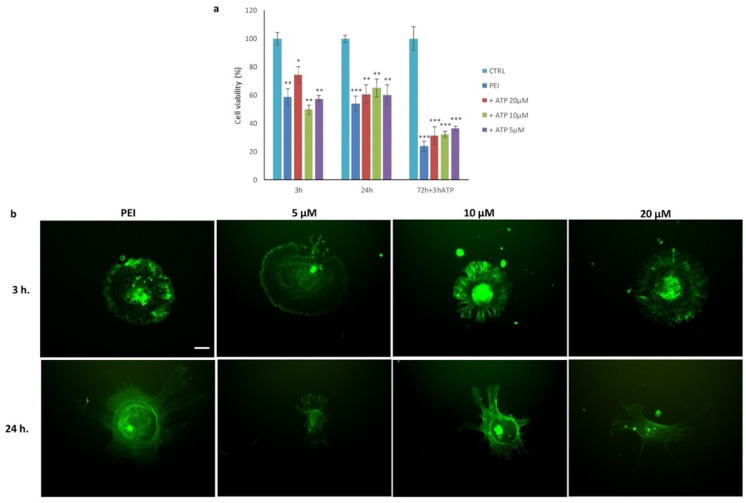
Effects of exogenous ATP on viability and microfilaments. (**a**) Values of the MTT assay are derived from the average of three experiments and expressed as percentages referring to the control; * *p* < 0.05, ** *p* < 0.01 and *** *p* < 0.001 versus CTRL (one-way ANOVA). (**b**) Representative images of actin cytoskeleton after 3 and 24 h of culture in medium supplemented with different concentrations of ATP; green represents FITC-conjugated antibody stain of FAK scale bar: 5 µm (for all the images shown).

The decrease in ATP shown in cells grown on the PEI-ended surface could be the cause of the dysfunctions of the numerous ATP-dependent cellular functions and of the entire cell metabolism. These low ATP levels of MSCs adhering to PEI-ended could be due to a destabilization of the plasma membrane that makes the mitochondrial component more accessible [40]. The alteration of the plasma membrane is via an SOS protein, which establishes a link between FAK activation and a well-established mitogenesis pathway. Furthermore, cell attachment via integrins controls the cellular sensitivity to the growth factor. This synergy between signals generated by cell adhesion receptors and the growth factor receptors helps to observe the SEM image shown in Figure 9b. The ΔΨ analysis, carried out in 3, 24, and 48 h (Figure 9c) with the fluorescent JC-1 dye, showed a decrease in mitochondrial activity in PEI samples, maybe due to an impairment of electron transport through the inner membrane coupled to proton pumping. From the observation under the fluorescence microscope, we noted a limited mitochondrial activity in PEI-ended samples in all experimental times analyzed and a greater concentration and distribution of mitochondria at the cytoplasmic level, with respect to the control cells spreading on the uncoated substrate. Given that the decreased mitochondrial activity does not permit the restoration of the intracellular ATP concentration necessary to reactivate a correct metabolic pathway, we studied the effect that extracellular ATP has on MSCs seeded on the PEI coating and supplemented the culture medium with different concentrations of ATP. After 72 h, we replaced the medium with fresh medium containing ATP (20 µM, 10 µM, and 5 µM) for 3 h and measured vitality by MTT (Figure 10a) and stained microfilaments with FITC-phalloidin (Figure 10b).

The initial addition of ATP slightly improved the condition of the cells with respect to the PEI control, but after 72 h (plus 3 h ATP) the decrease in viability was the same as for PEI. Actin was totally disarranged in all samples and appeared as large fluorescent spots, located particularly in the periphery of the cells. The different concentrations of ATP added to the culture medium caused only a slight increase with respect to the PEI control.

The presence of exogenous ATP led to an initial improvement in cellular conditions, supporting the hypothesis that the depletion of ATP prevents cells from carrying out their normal processes. The correct organization of the cytoskeleton is an ATP-dependent process. Previous studies showed that the integrity and rearrangement of the microfilament organization were closely related to the levels of ATP, which plays a key role in the polymerization of actin molecules, regulating the passage from their monomeric form (G actin) to their polymeric form (F-actin) [41,42]. The loss of ATP resulted in a rapid increase in the rigidity of the cytoskeleton; moreover, the addition of ATP immediately reversed this effect, suggesting that the stiffness of the cytoskeleton depends on ATP-dependent molecular transformations, such as the sliding of actomyosin filaments or chemical modifications of cytoskeletal proteins (phosphorylation) [43].

## 4. Conclusions

The understanding of the basic cell seeding phenomena is of paramount importance for effective tissue engineering strategies of clinical relevance. The present paper reports the study of the early adhesion events of undifferentiated MSCs on two different polyelectrolyte multilayers: one PSS-ended (anionic surface) and the other PEI-ended (cationic surface) layer.

It was found that cells seeded on the PSS-ended surface presented morphology behavior of the principal cytoskeleton components and of the adhesion molecules (integrins, pFAK, and pPXN) very similar to those of the mesenchymal cells seeded on a reference glass substrate. The pattern of adhesion and the evolution of focal adhesion plaques retrace the classic model known for these cells, implying the cytoskeletal protein translocation from the central area to the periphery of the cell. Furthermore, we observed that, after eight days, the cells growing on the PSS-ended surface were able to form a monolayer, even if a slight decrease in viability and cell cycle progression was observed. Therefore, these results suggested that the PSS-ended surface was substantially biologically compatible with undifferentiated MSCs.

On the other hand, the PEI-ended surface showed a decrease in MSC viability and inhibition of proliferation that were activated by an uncorrected assembly between adhesion molecules and the cytoskeleton. In particular, the absence of microfilaments or stress fibers appears to prevent the translocation of integrins, pFAK, and pPXN to form focal adhesion complexes. Cells that were not spread on the PEI-ended surface maintained, therefore, the initial round-shaped morphology. Furthermore, the alteration that the PEI-ended surface induces on the plasma membrane and the loss of ATP could be the reason for the failure of actin polymerization. The QCM-D results indicate that, already at the early stage of cell–surface interaction, the cell membrane and near-membrane cytoplasm have a different rigidity on the two different PEM substrates, with cell adhesion being remarkably stronger for PEI-ended than for PSS-ended ones. This supports the hypothesis that the cell spreading and the related cytoskeletal reorganization is strongly prevented on the PEI-ended surface, due to the strong adhesion force. On the contrary, the lower rigidity observed for cells on PSS appears connected to correct cell–material interactions, allowing proper cell spreading, which in turn may trigger the cascade of transduction events that promote later regulated growth, cell differentiation, and migration.

In summary, the change in the spreading capability of the cells onto different substrates, already at early steps of interaction, following the sequestering of the adhesion molecules in the initial cell–substrate contact area, appears to control the relative cytostatic effects.

## Figures and Tables

**Table 1:** Percentage of gated cells for each phase of the cell cycle (G0/G1, S, and G2/M) after 48 h and 6 days.

	G0/G1 (%)		S (%)		G2/M (%)	
	48 h	6 Days	48 h	6 Days	48 h	6 Days
CTRL	68.4	83.6	10.7	6.6	13.6	8.1
PSS	66.0	68.1	12.7	11.1	16.9	11.6
PEI	58.7	20.0	8.0	10.0	8.7	40.0

## Supplementary Materials

The following supporting information can be downloaded at: https://www.mdpi.com/article/10.3390/polym14132643/s1, Figure S1: Distribution of integrins α1β1 (red fluorescence) and actin (green fluorescence) in MSCs seeded for 6 h and 24 h.
