# Supplementary material for: Cytostatic Effects of Polyethyleneimine Surfaces on the Mesenchymal Stromal Cell Cycle
Source: Polymers (Basel). 2022 Jun 29;14(13):2643. doi: 10.3390/polym14132643 (PMC9269326; doi:10.3390/polym14132643)
Supplement: Supplementary file 1 [file polymers-14-02643-s001.zip › polymers-1731995-supplementary.pdf]

Supplementary Materials

Cytostatic effects of polyethyleneimine surfaces on the mesenchymal stromal cell cycle

Anna Alba[1], Giusy Villaggio[1], Grazia Maria Lucia Messina[2,*], Massimo Caruso[3], Concetta Federico[4], Maria Teresa Cambria[3], Giovanni Marletta[2] and Fulvia Sinatra[1,*]

1 Dipartimento di Scienze Biomediche e Biotecnologiche, sezione di Biologia e Genetica, University of Catania, Via S. Sofia, 65 95123 Catania, Italy; sinatra@unict.it (F.S.); a.alba83@gmail.com (A.A.); gvillaggio@unict.it (G.V.)

2 Laboratory for Molecular Surface and Nanotechnology (LAMSUN), Department of Chemical Sciences, University of Catania, Viale A. Doria, 6, 95125 Catania, Italy; gml.messina@unict.it (G.M.L.M.); gmarletta@unict.it (G.M.)

3 Dipartimento di Scienze Biomediche e Biotecnologie, sezione di Biochimica, University of Catania, Via S. Sofia, 65, 95123 Catania, Italy; cambrimt@unict.it (M.T.C.); mcaruso@unict.it (M.C.)

4 Dipartimento di Scienze Biologiche, Geologiche e Ambientali, University of Catania, Via Androne, 81, 95124 Catania, Italy; concetta.federico@unict.it

* Correspondence: gml.messina@unict.it GMLM (Tel. +39-0957385083); sinatra@unict.it F.S.

The α1ß1 integrin is more uniformly diffused in the central area of the cell body after 6 h of adhesion and no significant differences are found between the samples analyzed. In the last time examined, α1 spot like fluorescence appears more diffuse in the cytoplasm (Figure **S1**: d and e). While in the cells on the PEI coating it maintains a central position, probably due to the lower cellular distension (Figure **S1**: f)

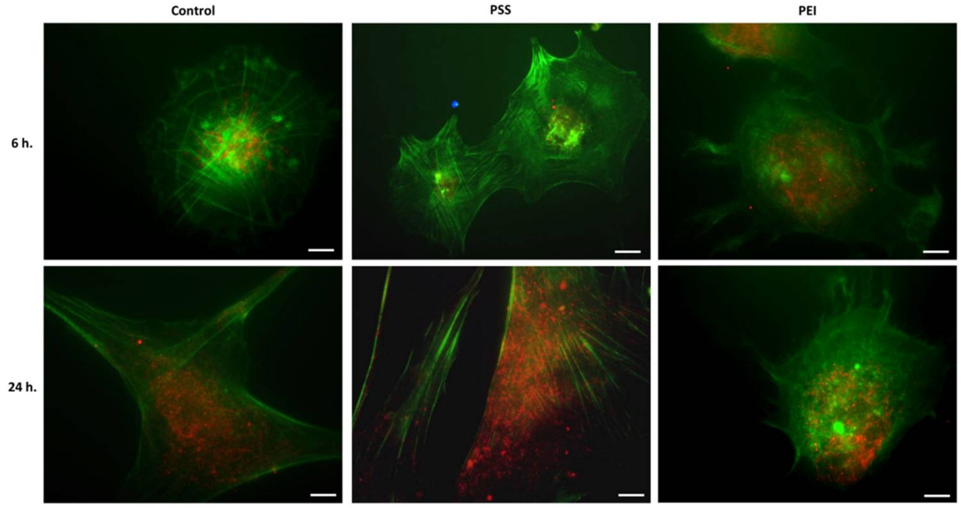

Figure S1: Distribution of integrins α1β1 (red fluorescence) and actin (green fluorescence) in MSC's seeded for 6h and 24 h on glass (a, d), PSS (b, e) and PEI (c, f) coated slides, a merge was made. Scale bar 2μm.
